# Development of SNP Genotyping Assays for Seed Composition Traits in Soybean

**DOI:** 10.1155/2017/6572969

**Published:** 2017-05-25

**Authors:** Gunvant Patil, Juhi Chaudhary, Tri D. Vuong, Brian Jenkins, Dan Qiu, Suhas Kadam, Grover J. Shannon, Henry T. Nguyen

**Affiliations:** ^1^Division of Plant Sciences, University of Missouri, Columbia, MO 65211, USA; ^2^Department of Agronomy and Genetics, University of Minnesota, St. Paul, MN 55108, USA

## Abstract

Seed composition is one of the most important determinants of the economic values in soybean. The quality and quantity of different seed components, such as oil, protein, and carbohydrates, are crucial ingredients in food, feed, and numerous industrial products. Soybean researchers have successfully developed and utilized a diverse set of molecular markers for seed trait improvement in soybean breeding programs. It is imperative to design and develop molecular assays that are accurate, robust, high-throughput, cost-effective, and available on a common genotyping platform. In the present study, we developed and validated KASP (Kompetitive allele-specific polymerase chain reaction) genotyping assays based on previously known functional mutant alleles for the seed composition traits, including fatty acids, oligosaccharides, trypsin inhibitor, and lipoxygenase. These assays were validated on mutant sources as well as mapping populations and precisely distinguish the homozygotes and heterozygotes of the mutant genes. With the obvious advantages, newly developed KASP assays in this study can substitute the genotyping assays that were previously developed for marker-assisted selection (MAS). The functional gene-based assay resource developed using common genotyping platform will be helpful to accelerate efforts to improve soybean seed composition traits.

## 1. Introduction

Soybean seed is an essential source of protein and edible oil for human diets and animal feed. Soybean seed contains about 40% protein and 21% oil on a dry matter basis and drives the economy of soybean industry. While seed oil and protein content are important, fatty acids (oil components) and amino acids (protein components) are desirable factors for long shelf life and nutrition [1]. Soybean oil typically contains high levels of linoleic and linolenic acids that lead to low oil stability and off-flavors with less functionality for industrial uses, limiting the commercial marketability of the soybean oil [2]. High oxidative stability is desirable for a longer shelf life of oil, which in turn depends on the presence of the monounsaturated fatty acid (oleic acid); thus, higher oleic acid and lower linolenic acid, without generating* trans*-fats, are desirable criteria [3, 4]. Another important component of soybean seeds is carbohydrates, of which concentration is essential for animal feed digestibility [5]. Soybean seeds contain about 15% carbohydrates that consist of sucrose (~6%), raffinose (~1%), and stachyose (~4%). Raffinose and stachyose accumulation is indigestible and causes flatulence in poultry, swine, and pets, thereby reducing the economic and dietary value of soybean seeds [6]. Similarly, lipoxygenase (Lpx) [7, 8] and Kunitz trypsin inhibitor (KTI) [9] are important components of soybean seed composition. Reduced content is preferred for reducing undesirable flavor and antinutritional properties. These traits are controlled by multiple homologous genes, which makes it more challenging for soybean lines with several beneficial seed traits.

Commodity soybean seed contains palmitic (11%), stearic (4%), oleic (23%), linoleic (54%), and linolenic acid (8%). Soybean oil with elevated oleic acid (>75%) is desirable for nutrition, flavor, and improvement of shelf life. Moreover, higher oleic acid content is desired in biodiesel production to improve the oxidative stability of oil while augmenting cold flow [10]. On the other hand, the higher concentration of polyunsaturated fatty acids (linolenic and linoleic) reduces the oxidative stability of the oil that decreases flavor and storage quality. Soybean oil used for cooking purposes is often partially hydrogenated, resulting in* trans*-fat in the oil, which is unhealthy for the heart. Several genetic studies have characterized genes controlling monounsaturated (oleic acid) and polyunsaturated fatty acids (linoleic and linolenic acid) in soybean [11–13]. During seed development stage, the two oleate desaturase genes, FAD2-1A* (Glyma10g42470)* and FAD2-1B* (Glyma20g24530)*, and the three linoleate desaturase genes, FAD3A* (Glyma14g37350)*, FAD3B* (Glyma02g39230)*, and FAD3C* (Glyma18g06950)*, are the major contributors controlling oleic and linolenic acid levels. The combination of mutant alleles of FAD2-1A and FAD2-1B produces up to 80% oleic acid in soybean seed [14]. Similarly, a combination of FAD3A, FAD3B, and FAD3C mutant alleles can produce ultralow (<1%) linolenic acid.

While high oleic and low linolenic acid content are of importance to the commodity soybean, the manipulation of raffinose family oligosaccharides (RFOs) is also an important aspect to improving soybean seed components [15]. In soybean, raffinose and stachyose are indigestible components that are undesirable in diets of poultry and swine [16]. The key step in raffinose and stachyose biosynthesis is mediated by the enzyme raffinose synthase (RS). Kerr and Sebastian (2000) reported PI 200508, with reduced levels of RFOs and elevated levels of sucrose due to variation in RS gene* (Glyma06g18890)*. Recently, Qiu et al. [17] reported a 33 bp deletion in the exon 4 of stachyose synthase gene (STS gene,* Glyma19g40550*) of PI 603176A having ultralow stachyose content (0.5%).

Soybean seed storage protein consists of approximately 6% proteinase inhibitors, Kunitz trypsin inhibitor (KTi, 21 kDa), and Bowman–Birk trypsin inhibitor (BBTi, 7-8 kDa), which also contributes to indigestibility [18]. Mutation in KTi gene* (Glyma08g45530)* prevents the accumulation of Kunitz trypsin inhibitor protein during seed development and improves tryptic activity [9].

In addition to undesirable traits mentioned above, the beany or grassy flavor of soybean seed products is a major factor that limits human consumption of soybean [19]. The grassy and beany flavor in soybean and other legumes seeds is due to the oxidation products resulting from seed lipoxygenase activity. Mature soybean seed consists of three distinct lipoxygenase isozymes, lipoxygenase 1* (Glyma13g42320)*, lipoxygenase 2* (Glyma13g42310)*, and lipoxygenase 3* (Glyma13g42330)*. Mutation in these genes leads to reduced lipoxygenase enzyme activity and results in better quality soybean meal [7, 8, 20].

In an effort to elevate oleic acid, soybean breeders are currently introgressing two mutant alleles of FAD2 and FAD3 genes into elite backgrounds to develop new soybean cultivars with elevated oleic and reduced linolenic acid (http://unitedsoybean.org/). To achieve this goal, a robust and high-throughput genotyping assay will play a vital role in marker-assisted selection (MAS) and marker-assisted backcrossing (MABC). Strongly associated functional markers improve selection of superior cultivars and reduce intensive phenotyping efforts [21–24]. For decades, different molecular marker technologies, such as randomly amplified polymorphic DNA (RAPD), simple sequence repeat (SSR), and diversity array technology [24], have been widely used in molecular plant breeding. However, these marker technologies are low-throughput, labor-intensive, and time-consuming compared with single nucleotide polymorphism (SNP) markers [23–28]. SNP markers have gained the popularity due to their robustness, suitability of automation, and abundance in the genomes; therefore, they are widely used in genetic diversity analysis, evolutionary relationships, and association mapping [29].

High-throughput genotyping platforms, such as Illumina GoldenGate arrays, Illumina Infinium BeadChips, Genotyping-by-sequencing, and Fluidigm SNPTypes, have been used for SNP genotyping of mapping populations and diverse germplasm. For improving seed composition in soybean, SimpleProbe and Taqman detection assays have been developed to identify mutant alleles of those genes [7, 14, 30–34]. A number of molecular markers (simple sequence repeats (SSR), randomly amplified polymorphic DNA (RAPD), sequence characterized amplified regions (SCAR), cleaved amplified polymorphic sequences (CAPS/dCAPS), and diversity array technology (DArT)) and genotyping assay have been used in molecular plant breeding over the last several decades [35, 36]. Although some of markers and genotyping platforms are useful for rapid genotyping and are codominant, they are not generally economical for carrying out large scale genotyping projects. For example, these assays may require additional restriction digestion steps followed by gel running [36]. Moreover, these assays may require comparatively higher quantity and quality of DNA, longer processing time, and additional optimization to obtain definite genotyping results [37, 38]. To choose a genotyping platform, several factors, such as accuracy, reproducibility, multiplexing, cost-effectiveness (cost per genotype and instrument running cost), and time required for optimization and assay-run, need to be considered. The KASP assays (KBioscience, Hoddesdon, UK) have emerged as novel cost-effective marker assays, especially for molecular breeding, which had many applications in several crop plants, including soybean [22, 23, 33, 34, 39–44]. Since the advent of KASP assays, it has been well adopted and employed in various crop breeding programs because of its advantages over other assays. These advantages include throughput, robustness, and cost-effectiveness coupled with the requirements of less DNA quality and concentration, easy sample preparation, and simple PCR running protocol. Thus, in view of the KASP assay advantages, the objective of the present study was to develop high-throughput, breeder-friendly, locus specific, and cost-effective KASP assays to detect SNP markers associated with the mutant alleles for soybean seed composition traits. The KASP assays validated in this article will be beneficial for germplasm characterization, allele mining, marker-assisted backcrossing, and marker-assisted recurrent selection [45] in soybean breeding programs.

## 2. Materials and Methods

### 2.1. Plant Materials

#### 2.1.1. Fatty Acids

A set of populations obtained from biparental crosses was used for validation of oleic and linolenic acid assays (Supplementary Table S1 in Supplementary Material available online at https://doi.org/10.1155/2017/6572969). In addition, leaf tissue and FTA cards (Whatman Inc., Clifton, NJ, USA) were (at V2 stage) collected to validate FAD2-1B (MU-HO-4) genotyping assay. FTA is a paper-based system designed to fix and store nucleic acids directly from fresh tissues pressed into the treated paper (Fehr and Caviness, 1997 [51]).

#### 2.1.2. Carbohydrates

For raffinose synthase (MU-RS2-1 and MU-RS2-2) assays, mutant sources (PI 200508 and mutagenized line 397) and artificial heterozygotes were tested along with a wild-type genotype, cultivar Williams 82. For stachyose synthase assay (MU-STS-1), an F2 population was used for marker validation [17].

#### 2.1.3. Trypsin Inhibitor and Lipoxygenase

An F_2_ population SP6A-209 X PI 542044 was used to test for a Kunitz trypsin inhibitor assay (MU-KTI-1). SP6A-209 was F5 line carrying* sts* mutant allele with low stachyose and PI 542044 was germplasm with null trypsin inhibitor (KTI). Due to the unavailability of a segregating population for lipoxygenase assays (MU-Lox-1, MU-Lox-2, and MU-Lox-3), the mutant sources PI 408251 [7], Jinpumkong-2 [8] and PI 205085, and PI 417458 [7] were grown in a greenhouse and tested, respectively, along with a nonmutant genotype, cultivar Williams 82 (Table 1).

Young leaf tissue of V1 soybean seedlings was collected and freeze-dried for genomic DNA extraction. The tissue was ground using GenoGrinder (SPEX CertiPrep, Metuchen, NJ, USA) in a microcentrifuge tube at 1,600 rpm for 2 min. DNA extraction was then performed following a nonhazardous protocol as previously described by King et al. (2014).

### 2.2. KASP Assay Development

To develop* KASP* genotyping assays, sequence information based on published mutant genes and sources (Table 1) was used [7, 14, 17, 31, 32, 47]. The* KASP* genotyping assay is fluorescence (FRET) based assay and enables identification of biallelic SNPs and InDels. Two allele-specific forward primers along with tail sequences and one common reverse primer (Table 2) were synthesized by LGC Genomics (http://www.lgcgroup.com). The reaction mixture was prepared following the manufacturer's instructions with minor modifications in number of cycles (KBioscience; http://www.lgcgroup.com/products/kasp-genotyping-chemistry/#.VsZK7PkrKM8). Briefly, KASP assays were run with 10 *μ*L final reaction volume containing 5 *μ*L KASP master mix, 0.14 *μ*L primer mix, 2 *μ*L of 10–20 ng/*μ*L genomic DNA, and 2.86 *μ*L of water. The following thermal cycling conditions were used: 15 min at 95°C, followed by 10 touchdown cycles of 20 s at 94°C and 1 min at 61–55°C (dropping 0.6°C per cycle), and then 26 cycles of 20 s at 94°C and 1 min at 55°C. For each assay 26 cycles were used excluding MU-HO-4 and MU-LL-1 which required additional 4 cycles (total 30 cycles). The fluorescent endpoint genotyping method was carried out using Roche LightCycler 480-II instrument (Roche Applied Sciences, Indianapolis, IN, USA). In endpoint genotyping a signal is generated when dyes bound to allele-specific probes and depending upon hybridization (mismatch or perfect match) the corresponding fluorescent single can be measured (https://molecular.roche.com/systems/lightcycler-480-system/).

## 3. Results and Discussion

Several breeding programs in the US are currently in the process of introgressing fatty acids and carbohydrates (CHO) mutant genes into soybean cultivars with elevated oleic and reduced linolenic acids along with modified CHO. Soybean varieties with desirable seed composition traits will help increase soybean demand and benefit the soybean industry. A robust and cost-effective genotyping assay is of prime importance to combine these desirable genes using MABC. In previous studies, the genetic basis of high oleic and low linolenic acid content has been identified in certain sources and functional SNP assays have also been developed [13, 30, 32, 48, 49, 52]. However, the existing SNP genotyping assays are not as breeder-friendly for applications in the soybean breeding programs with high-throughput settings. Recently, Shi et al. [33, 34] developed SNP assays for fatty acid mutants using different chemistry and suggested the advantage of those assays related to processing time and DNA quality [33, 34]. Previous assays for fatty acid mutants were developed using either SimpleProbe or TaqMan chemistry. As different genetic markers are frequently applied for germplasm or population screening, there is need for common genotyping platform. The combination of genotyping assays with different chemistry (e.g., SimpleProbe, TaqMan, or KASP) is being used in soybean breeding program; however utilization of different type assays in one breeding program may not be as efficient in terms of handling, data analysis, and variation in protocol. In this study, all the assays were developed using a common genotyping chemistry and platform, KASP technology, which could minimize common laboratory errors, such as handling different reaction mixtures, protocols for assays run, data analysis, and data export.

We have successfully developed and validated KASP assays to detect functional SNP or Indel markers for previously reported genes related to various seed composition traits. Sequence information of target genes and corresponding mutant sources are summarized in Table 1. The data obtained in the present study was compared with the previously reported SimpleProbe and Taqman assays (Supplementary Tables S2, S3, and S4). Initially, some of the KASP assays did not show a clear separation of heterozygote alleles from either mutant or wild-type alleles. Subsequently, this problem was overcome by optimization, in which additional numbers of thermal cycles were added.

**Figure 1:**
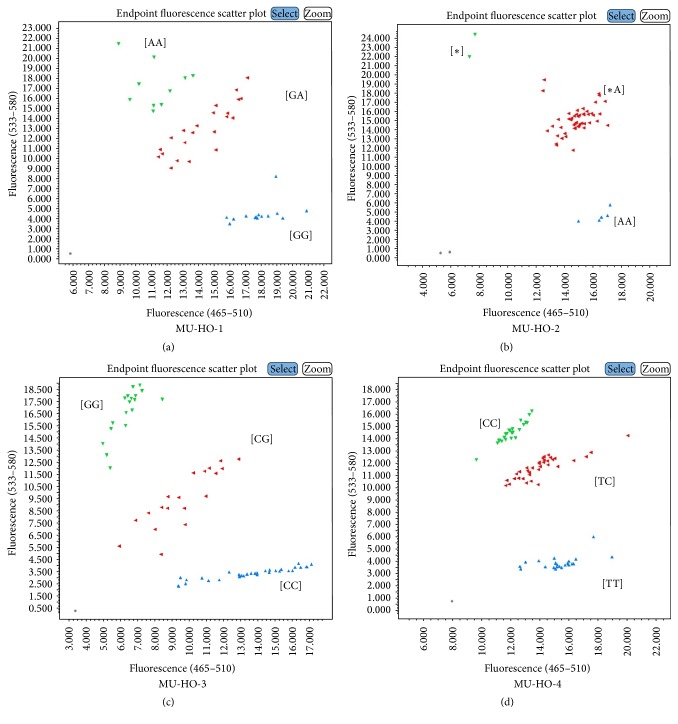
Endpoint fluorescence genotyping plots for high oleic (HO) acid trait. (a) FAD2-1A_17D where [GG] is wild-type with normal oleic acid content and [AA] is mutant with elevated oleic acid content; (b) FAD2-1A_Del where [AA] is wild-type with normal oleic acid content and [*∗*] is mutant with elevated oleic acid content; (c) FAD2-1B_327 where [CC] is wild-type with normal oleic acid content and [GG] is mutant with elevated oleic acid content; (d) FAD2-1B_I143T where [TT] is wild-type with normal oleic acid content and [CC] is mutant with elevated oleic acid content.

**Figure 2:**
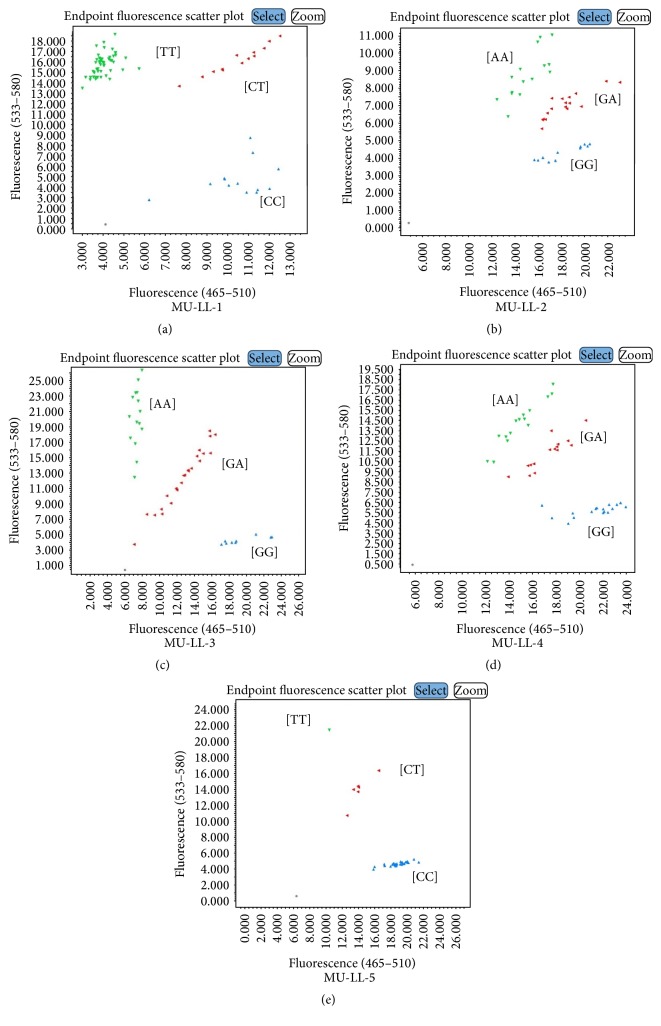
Endpoint fluorescence genotyping plots for trait for low linolenic (LL) acid trait. (a) FAD3A_Del where [TT] is wild-type with normal linolenic acid content and [CC] is mutant with reduced linolenic acid content; (b) FAD3A_Splice where [GG] is wild-type with normal linolenic acid content and [TT] is mutant with reduced linolenic acid content; (c) FAD3B_Splice where [GG] is wild-type with normal linolenic acid content and [AA] is mutant with reduced linolenic acid content; (d) FAD3C_G128E where [GG] is wild-type with normal linolenic acid content and [AA] is mutant with reduced linolenic acid content; (e) FAD3C_H304Y where [CC] is wild-type with normal linolenic acid content and [TT] is mutant with reduced linolenic acid content.

Among several genotyping assays tested, the assays of FAD2-1A gene for two mutant sources, 17D (MU-HO-1) and a deletion mutant, PI 603452 (MU-HO-2), were validated in segregating populations (Supplementary Table S2; Figures 1(a) and 1(b)). Similarly, the genotyping assays developed to detect SNP markers of two mutants of FAD2-1B [PI 283327 (MU-HO-3) and PI 578451 (MU-HO-4)] were successfully validated (Figures 1(c) and 1(d)). To access the robustness of these genotyping assays, we used FTA cards (Whatman Inc., Clifton, NJ, USA) and tested with MU-HO-4 assays. The analysis showed a perfect correlation for the assays tested with genomic DNA (data not shown). The results were compared with previously reported molecular marker assays for validation [32, 46, 47].* For linolenic acid*, MU-LL-1, MU-LL-2, MU-LL-3, MU-LL-4, and MU-LL-5 assays were developed and tested along with the SimpleProbe and the TaqMan assay methods in the segregating populations (Supplementary Table S3; Figure 2). A strong correlation and a clear separation of three genotypic clusters for MU-LL-1 (97.5%), MU-LL-2 (93%), MU-LL-3 (98%), MU-LL-4 (100%), and MU-LL-5 (95%) were observed. Recently, Pham et al. [31] characterized the* fan1* locus in a soybean line A5 and developed a TaqMan assay for a complex insertion and deletion in the FAD3A gene. Using a PCR-based genomic strategy, they identified a 6.4 kbp deletion and 165 bp insertion in the FAD3A gene. We utilized the sequence information to design a forward, fluorescein amidite (FAM) labelled primer (specific to the 165 bp insertion) and hexachloro-fluorescein (HEX) labelled primers (specific to the 6.4 kbp deletion; Williams 82) along with a common reverse primer. A strong correlation (98%) with a previously designed Taq-man assay [31] was observed (Supplementary Table S3).

**Figure 3:**
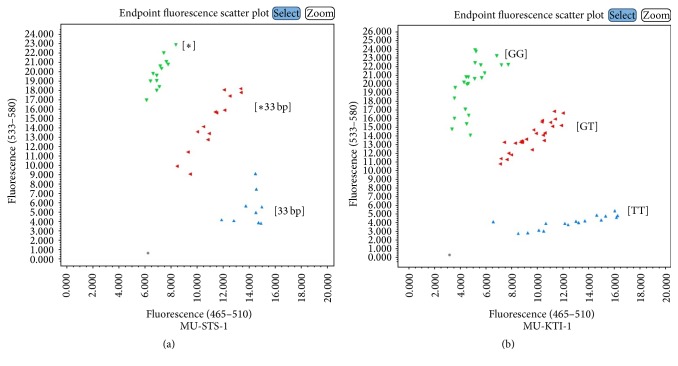
Endpoint fluorescence genotyping plots for trait for (a) stachyose synthase (STS) where [*∗*] is mutant allele with reduced stachyose and [33 bp] is wild-type allele with normal stachyose concentration; (b) Kunitz trypsin inhibitor (KTI) where [GG] is wild-type allele with normal trypsin and [TT] is mutant allele with reduced trypsin content.

**Figure 4:**
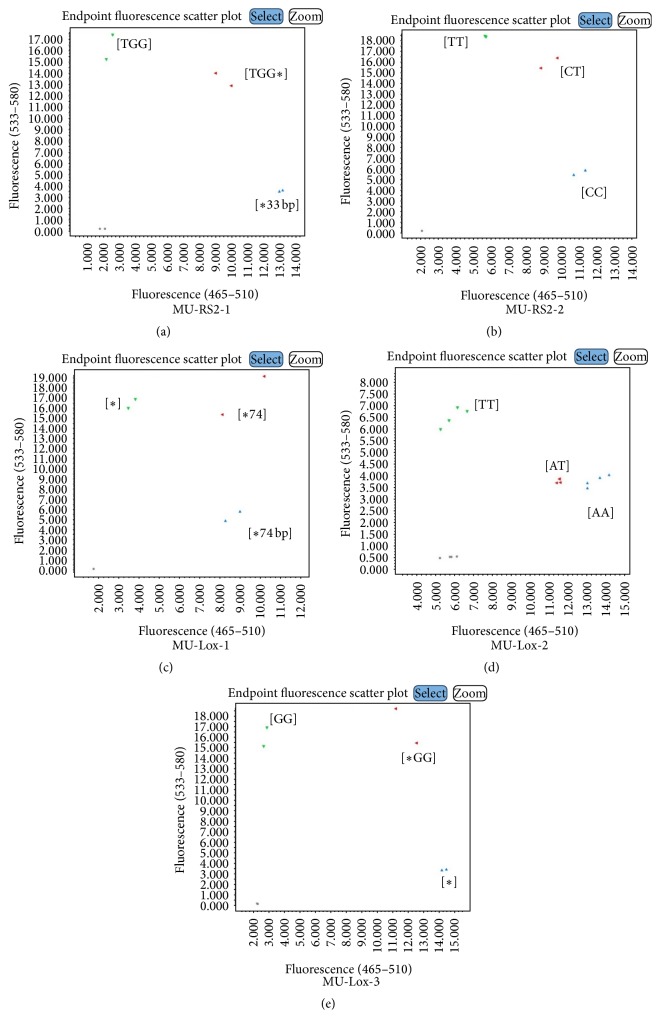
Endpoint fluorescence genotyping plots for raffinose synthase (RS2) and lipoxygenase (Lox). (a) RS2_W where [TGG] is wild-type allele with normal raffinose and [*∗*3 bp] is mutant allele with reduced raffinose; (b) RS2_397 where [CC] is wild-type allele with normal raffinose and [TT] is mutant allele with reduced raffinose; (c) Lox-1 where [*∗*] is wild-type with normal lipoxygenase and [*∗*74] is mutant allele with reduced lipoxygenase; (d) Lox-2 where [TT] is wild-type allele and [AA] is mutant allele with reduced lipoxygenase; (e) Lox3_G_100 where [GG] is wild-type allele and [*∗*] is mutant allele with reduced lipoxygenase.

The KASP assays were also developed and validated for mutant genes conferring traits important for soybean meal digestibility, which included raffinose synthase (rs2), stachyose synthase (sts), and Kunitz trypsin inhibitor (kti). The MU-STS-1 and MU-KTI-1 assays were tested in a genetic mapping population and found to be in concordance with earlier reported molecular marker assays [17]. The MU-STS-1 assay showed a 78% of correlation with previously developed genotyping assay, while MU-KTI-1 showed 98.4% correlation (Supplementary Table S4, Figures 3(a) and 3(b)). Due to the unavailability of mapping populations or diverse germplasm for MU-RS2-1, MU-RS2-2, and MU-Lox-1-3, these markers were tested on corresponding mutant and wild-type (Williams 82) sources. All assays accurately detected desired genotypes (Figures 4(a)–4(e)). We also tested several assays on artificial heterozygous genomic DNA, in which the DNA of mutant and wild-type (Williams 82) lines were mixed at equal concentrations of 10 ng/*μ*L. This artificial heterozygote allele correctly designated the genotype and was clustered between mutant and wild-type alleles (Figures 4(a)–4(e)).

The validated KASP detection assays in our study will be beneficial for various practical applications, such as germplasm characterization, allele mining, and MAS in backcrossing and forward breeding programs. These SNP genotyping assays can be used for both routinely extracted genomic DNA and FTA cards (for DNA collection) to screen genetic and breeding materials at early growth stages (V1 or V2) (Fehr and Caviness, 1997). The DNA obtained from FTA card and assayed using KASP is a rapid and more economical screening compared to DNA extracted using other methods. The chemistry of KASP assays offered flexibility in designing and high locus specificity, which makes the assays accurate and robust. Unlike other PCR-based genotyping assays, KASP requires no labelling of the target-specific primer or probes, which provide cost advantage over other genotyping assays. The KASP assay is specific to the SNP or Indel markers to be targeted and consists of two competitive, allele-specific forward primers (labelled with fluorescent dye) and one common primer (http://www.lgcgroup.com/). In addition, the clustering of data points from the assay allows the genotyping results to be straightforward, which greatly benefits the SNP allele identification.

To estimate the economic profile, the costs of KASP assays were compared with SimpleProbe and Taqman assay (Supplementary Table S5). The comparison showed that total costs of the KASP assays were the most economical among the three tested assays (Supplementary Table S5). For instance, the costs of KASP assays were about 35% of those of TaqMan assays and about 70% of those of SimpleProbe assays. Moreover, if the KASP assay is optimized for the 5 *μ*L of reaction volume, the cost of the assay is likely to be even more economical compared to other assays. The small reaction volume, simplicity of PCR implementation, and data acquisition will make the entire genotyping process affordable, fast, and accurate. Because of these advantages, KASP assays have been developed for various traits in soybean, such as frogeye leaf spot resistance [53], soybean cyst nematode resistance [22, 33, 34], and salinity tolerance [23].

In summary, the improvement of soybean seed composition traits is of tremendous interest to soybean growers because of its nutritional importance in food and feed as well as industrial applications. This study successfully developed robust and cost-effective KASP genotyping assays based on previously identified functional SNP marker information for six seed composition traits. It included high oleic, low linolenic acid, raffinose and stachyose synthase, Kunitz trypsin inhibitor, and lipoxygenase traits. It remarkably demonstrated robustness, high-throughput, and cost-effectiveness of KASP genotyping assays. Moreover, the accuracy of the assays was also validated in multiple soybean breeding populations and by data comparison with other genotyping assays; thus, the assays developed on a common genotyping platform can be adopted in soybean breeding programs, which facilitate the MAS of seed composition traits with greater efficiency and accuracy.

## Supplementary Material

Supplementary Table 1: lists plant material used in this study and is self-explanatory.
Supplementary Tables 2–4: lists compares genotype call and it is explained in the main-text.
Supplementary Table 5: also explained in text.

## Figures and Tables

**Table 1:** A summary of mutant sources of SNP markers and KASP genotyping assays developed and validated in various genetic materials for different seed composition traits used in the present study.

Trait	Marker	Assay ID	Gene	Position	Effect on trait	Donor checks	Reference
Oleic acid	FAD2-1A_17D	MU-HO-1	*Glyma10g42470*	S117N	Improves oil shelf life	17 D	[46]
	FAD2-1A_A_Del	MU-HO-2		^*∗*^A544		PI603452	[47]
	FAD2-1B_327	MU-HO-3	*Glyma20G24530*	P137R		PI283327	[32]
	FAD2-1B_I143T	MU-HO-4		I143T		PI578451	[32]

Linolenic acid	FAD3A_Del	MU-LL-1	*Glyma14g37350*	6.4 kb del.	Oxidative and flavor instability	IA3024	[31]
	FAD3A_Splice	MU-LL-2		G810A		CX1512-44; SB-01	[48]
	FAD3B_Splice	MU-LL-3	*Glyma02g39230*	G462A		IA3024	[49]
	FAD3C_G128E	MU-LL-4	*Glyma18g06950*	G128E		CX1512-44; SB-01	[48]
	FAD3C_H304Y	MU-LL-5		H304Y		A29, IA3024	[49]

Kunitz trypsin inhibitor	KTI	MU-KTI-1	*Glyma08g45530*	Q120^*∗*^	Antinutritional	PI 542044	[9]

Stachyose synthase	STS	MU-STS-1	*Glyma19g40550*	33 bp del.	Antinutritional	SP6A-209	[17]

Raffinose synthase	RS2_W	MU-RS2-1	*Glyma06g18890*	^*∗*^TGG991	Antinutritional	PI 200508	[50]
	RS2_397	MU-RS2-2		T107I	Antinutritional	397	[46]

Lipoxygenase	Lox-1	MU-Lox-1	*Glyma13g42320*	74 bp del.	Beany flavor	PI 408251	[7]
	Lox-2	MU-Lox-2	*Glyma13g42310*	H532Q	Beany flavor	Jinpumkong-2	[8]
	Lox3_G_100	MU-Lox-3	*Glyma15g03030*	^*∗*^G100	Beany flavor	PI 205085; PI 417458	[7]

*∗*: deletion.

**Table 2:** A list of SNP markers, designated ID, primers, and probes for KASP assays developed and validated in in this study.

Marker	Fluorescent primer	Sequence
FAD2-1A_17D (MU-HO-1)	FAM_primer	TCTCACTGGTGTGTGGGTGATTGCTCACGAGTGTGGTCACCATGCCTTCA***G***
	HEX_primer	TCTCACTGGTGTGTGGGTGATTGCTCACGAGTGTGGTCACCATGCCTTCA**A**
	Reverse primer	CAAGTACCAATGGGTTGATGATGTTGTGGGTTTGACCCTTCACTCAACA

FAD2-1A_452 (MU-HO-2)	FAM_primer	TTTGTCCCAAAACCAAAATCCAAAGTTGCATGGTTTTCCAAGTACTTAAACAACCCTCTAGGA***A***
	HEX_primer	TTTGTCCCAAAACCAAAATCCAAAGTTGCATGGTTTTCCAAGTACTTAAACAACCCTCTAGGA _^*∗*^
	Reverse primer	GGGCTGTTTCTCTTCTCGTCACACTCACAATAGGGTGGCCTATGTATTTAGCCTTC

FAD2-1B_327 (MU-HO-3)	FAM_primer	CCTTCAGCAAGTACCCATGGGTTGATGATGTTATGGGTTTGACCGTTCACTCAGCACTTTTAGTCC***C***
	HEX_primer	CCTTCAGCAAGTACCCATGGGTTGATGATGTTATGGGTTTGACCGTTCACTCAGCACTTTTAGTCC**G**
	Reverse primer	TTATTTCTCATGGAAAAYAAGCCATCGCCGCCACCACTCCAACACGGGTTCCCTTGACCGTGATG

FAD2-1B_I143T (MU-HO-4)	FAM_primer	TGGGTTGATGATGTTATGGGTTTGACCGTTCACTCAGCACTTTTAGTCC**S**TTATTTCTCATGGAAAA***T***
	HEX_primer	TGGGTTGATGATGTTATGGGTTTGACCGTTCACTCAGCACTTTTAGTCC**S**TTATTTCTCATGGAAAA**C**
	Reverse primer	AAGCCATCGCCGCCACCACTCCAACACGGGTTCCCTTGACCGTGATGAAGTGTTTGTCC

FAD3A_Del (MU-LL-1)	FAM_primer	AATAAGAACTAAATTTAAAAGTGAAGTTGAA*T*
	HEX_primer	AATAAGAACTAAATTTAAAAGTGAAGTTGAAC
	Reverse primer	GCAAGTTGTTCAGAAAACGTTAAAACTCC

FAD3A_Splice (MU-LL-2)	FAM_primer	CTGGACTTTGTCACATACTTGCATCACCATGGTCATCATCAGAAACTGCCTTGGTATCGCGGCAAG***G***
	HEX_primer	CTGGACTTTGTCACATACTTGCATCACCATGGTCATCATCAGAAACTGCCTTGGTATCGCGGCAAG**A**
	Reverse primer	TAACAAAAATAAATAGAAAATAGTGAGTGAACACTTAAATGTTAGATACTACCTTCTTCTTCTTT

FAD3B_Splice (MU-LL-3)	FAM_primer	GAATCTTTATGCTTCCTGAGGCTGTTCTTGAACATGGCTCTTTTTTATGTGTCATTATCTTA***G***
	HEX_primer	GAATCTTTATGCTTCCTGAGGCTGTTCTTGAACATGGCTCTTTTTTATGTGTCATTATCTTA**A**
	Reverse primer	TTAACAGAGAAGATTTACAAGAATCTAGACAGCATGACAAGACTCATTAGATTCACTGT

FAD3C_G128E (MU-LL-4)	FAM_primer	CATGGAAGTTTTTCAAACAGTCCTTTGTTGAACAGCATTGTGGGCCACATCTTGCACTCTTCAATTCTTGTACCATACCATG***G***
	HEX_primer	CATGGAAGTTTTTCAAACAGTCCTTTGTTGAACAGCATTGTGGGCCACATCTTGCACTCTTCAATTCTTGTACCATACCATG**A**
	Reverse primer	ATGGTCGGTTCCTTTTAGCAACTTTTCATGTTCACTTTGTCCTTAAATTTTTTTTTATGTTTGTT

FAD3C_H304Y (MU-LL-5)	FAM_primer	TAGATCGCGACTATGGTTGGATCAACAACATTCACCATGACATTGGCACCCATGTTATCCAT***C***
	HEX_primer	TAGATCGCGACTATGGTTGGATCAACAACATTCACCATGACATTGGCACCCATGTTATCCAT**T**
	Reverse primer	ACCTTTTCCCTCAAATTCCACATTATCATTTAATCGAAGCGGTATTAATTCTCTATTTC

KTI_2 (MU-KTI-1)	FAM_primer	AACAAAGATGCAATGGATGGTTGGTTTAGACTT*G*
	HEX_primer	AACAAAGATGCAATGGATGGTTGGTTTAGACTTT
	Reverse primer	AGAGNNNNNNNTTTCTGATGATGAATTCAATAACTAT

STS_2 (MU-STS-1)	FAM_primer	GAATTGGAGTGTGTTGAGAAGGGTGCAAAGGTTAAGGTTAAGGGTGATGGGAGATTCCTT_^+^
	HEX_primer	GAATTGGAGTGTGTTGAGAAGGGTGCAAAGGTTAAGGTTAAGGGTGATGGGAGATTCCTT**GCTTACTCAAGTGAATCTCCAAAGAAGTTCCAAY**
	Reverse primer	TGAATGGTTCTGATGTTGCTTTTGAGTGGCTCCCTGATGGAAAACTCACTCTCAACCTTGCTTGGATTGAAGAGAATGGCGGGGTT

RS2_W (MU-RS2-1)	FAM_primer	GGTATGGGTGCCTTTGTTAGGGACTTGAAGGAACAGTTTAGGAGCGTGGAGCAGGTGTATGTG_^+^
	HEX_primer	GGTATGGGTGCCTTTGTTAGGGACTTGAAGGAACAGTTTAGGAGCGTGGAGCAGGTGTATGTG**TGG**
	Reverse primer	CACGCGCTTTGTGGGTATTGGGGTGGGGTCAGACCCAAGGTTCCGGGCATGCCCCAGGCTAAGGTT

RS2_397 (MU-RS2-2)	FAM_primer	CTGGGGAAGCTCAGAGGAATAAAATTCATGAGCATATTCCGGTTTAAGGTGTGGTGGACCA***C***
	HEX_primer	CTGGGGAAGCTCAGAGGAATAAAATTCATGAGCATATTCCGGTTTAAGGTGTGGTGGACCA**T**
	Reverse primer	TCACTGGGTCGGTAGCAACGGACACGAACTGGAGCACGAGACACAGATGATGCTTCTCGACA

Lox-1 (MU-Lox-1)	FAM_primer	CATGCGGCGATGGAGCCATTCGTCATAGCAACACACCGACATCTTAGCGTGCTTCACCCAATTTA_^+^
	HEX_primer	CATGCGGCGATGGAGCCATTCGTCATAGCAACACACCGACATCTTAGCGTGCTTCACCCAATTTA**CAAGCTTCTGACTCCTCACTATCGTAACAACATGAACATCAACGCACTTGCCAGGCAATCTCTAATTAATGCTA**
	Reverse primer	ATGGCATAATAGAGACAACCTTTTTGCCCTCAAAGTATTCTGTGGAGATGTCTTCGGCGGTTT

Lox-2 (MU-Lox-2)	FAM_primer	CATCTATGATGTATGTTATGTCTCAATTTTATTTTATTTTTATTTTTTATTTTGTTCATAGGTTAAATACTCA***T***
	HEX_primer	CATCTATGATGTATGTTATGTCTCAATTTTATTTTATTTTTATTTTTTATTTTGTTCATAGGTTAAATACTCA**A**
	Reverse primer	GCGGTGATTGAGCCATTCATCATAGCAACAAACCGACACCTTAGTGCTCTTCACCCAATTTATAAGCTTC

Lox3_G_100 (MU-Lox-3)	FAM_primer	AGGGACAGTGGTGTTGATGCGCAAGAATGTGTTGGACGTGAATAGCGTAACCAGCGTTGGGG_^+^
	HEX_primer	AGGGACAGTGGTGTTGATGCGCAAGAATGTGTTGGACGTGAATAGCGTAACCAGCGTTGGGG**G**
	Reverse primer	AATTATTGGTCAAGGTCTCGACTTAGTTGGCTCAACACTCGATACTCTTACTGCCTT

_^*∗*^: HEX_primer and _^+^: FAM_primer.
